# Ecto-5’-Nucleotidase Overexpression Reduces Tumor Growth in a Xenograph Medulloblastoma Model

**DOI:** 10.1371/journal.pone.0140996

**Published:** 2015-10-22

**Authors:** Angélica R. Cappellari, Micheli M. Pillat, Hellio D. N. Souza, Fabrícia Dietrich, Francine H. Oliveira, Fabrício Figueiró, Ana L. Abujamra, Rafael Roesler, Joanna Lecka, Jean Sévigny, Ana Maria O. Battastini, Henning Ulrich

**Affiliations:** 1 Programa de Pós-Graduação em Ciências Biológicas: Bioquímica, Instituto de Ciências Básicas da Saúde, Universidade Federal do Rio Grande do Sul, Porto Alegre, RS, Brazil; 2 Departamento de Bioquímica, Instituto de Ciências Básicas e da Saúde, UFRGS, Porto Alegre, RS, Brazil; 3 Departamento de Bioquímica, Instituto de Química, Universidade de São Paulo, São Paulo, SP, Brazil; 4 Serviço de Patologia, Hospital de Clínicas de Porto Alegre, UFRGS, Porto Alegre, RS, Brazil; 5 Laboratório de Pesquisa em Câncer, Hospital de Clínicas de Porto Alegre, UFRGS, Porto Alegre, RS, Brazil; 6 Instituto do Câncer Infantil do RS, ICI-RS, Porto Alegre, RS, Brazil; 7 Departamento de Farmacologia, Instituto de Ciências Básicas da Saúde, UFRGS, Porto Alegre, RS, Brazil; 8 Instituto Nacional de Ciência e Tecnologia Translacional em Medicina, UFRGS, Porto Alegre, RS, Brazil; 9 Département de microbiologie-infectiologie et d’immunologie, Faculté de Médecine, Université Laval, Québec, G1V 0A6, QC, Canada; 10 Centre de recherche du CHU de Québec, Québec, G1V 4G2, QC, Canada; University of Navarra, SPAIN

## Abstract

**Background:**

Ecto-5’-nucleotidase/CD73 (ecto-5’-NT) participates in extracellular ATP catabolism by converting adenosine monophosphate (AMP) into adenosine. This enzyme affects the progression and invasiveness of different tumors. Furthermore, the expression of ecto-5’-NT has also been suggested as a favorable prognostic marker, attributing to this enzyme contradictory functions in cancer. Medulloblastoma (MB) is the most common brain tumor of the cerebellum and affects mainly children.

**Materials and Methods:**

The effects of ecto-5’-NT overexpression on human MB tumor growth were studied in an *in vivo* model. Balb/c immunodeficient (nude) 6 to 14-week-old mice were used for dorsal subcutaneous xenograph tumor implant. Tumor development was evaluated by pathophysiological analysis. In addition, the expression patterns of adenosine receptors were verified.

**Results:**

The human MB cell line D283, transfected with ecto-5’-NT (D283hCD73), revealed reduced tumor growth compared to the original cell line transfected with an empty vector. D283hCD73 generated tumors with a reduced proliferative index, lower vascularization, the presence of differentiated cells and increased active caspase-3 expression. Prominent A_1_ adenosine receptor expression rates were detected in MB cells overexpressing ecto-5’-NT.

**Conclusion:**

This work suggests that ecto-5’-NT promotes reduced tumor growth to reduce cell proliferation and vascularization, promote higher differentiation rates and initiate apoptosis, supposedly by accumulating adenosine, which then acts through A_1_ adenosine receptors. Therefore, ecto-5’-NT might be considered an important prognostic marker, being associated with good prognosis and used as a potential target for therapy.

## Introduction

Ecto-5’-nucleotidase/CD73 (ecto-5’-NT) is expressed by various human tissues and considered the main producer of extracellular adenosine [1]. Adenosine activates P1 metabotropic receptors, subdivided into A_1_, A_2A_, A_2B_ and A_3_ receptors, which participate in the control of intracellular cAMP levels [2]. Ecto-5’-NT influences cancer progression in different types of tumors, including bladder and breast cancer, melanomas and gliomas [1]. Sadej and co-workers (2006) [3] demonstrated that ecto-5’-NT expression increased with the degree of malignancy of human melanoma cell lines, where higher expression levels were measured in a metastatic melanoma cell line. In breast cancer, the involvement of ecto-5’-NT in invasiveness and its interaction with extracellular matrix proteins were demonstrated [4]. Previous studies from our laboratory have shown a role of ecto-5’-NT in glioma progression and tissue invasiveness events. First, different glioma cell lines expressed prominent levels of ecto-5’-NT compared to normal astrocytes [5]. Second, increased cellular confluence was accompanied by enhanced ecto-5’-NT expression and activity [6]. Third, diminished ecto-5’-NT activity affected glioma cell adhesion and reduced cell proliferation [7, 6], suggesting the importance of ecto-5’-NT enzymatic activity for glioma cell survival.

However, immunohistochemistry and microarray analysis of human breast cancer samples revealed that ecto-5’-NT overexpression, which was observed in 74% of analyzed tissues, was correlated with the disease-free state and overall survival, suggesting that the expression of this enzyme is associated with good prognosis [8]. Ecto-5’-NT expression levels in medulloblastoma (MB) cell lines were reported in our previous paper. While the primary MB cell lines (Daoy and ONS76) expressed this enzyme, the metastatic MB cell line (D283) did not [9]. This difference was attributed to the regulation of ecto-5’-NT expression by β-catenin nuclear immunoreactivity [10], which has been suggested to predict a favorable prognostic for MB [11]. Unlike gliomas, MB mainly affects children with a median age of 9 years, and the median survival of the patient is approximately 5 years [12]. These tumors occur preferentially in the cerebellum and are considered the most common brain tumors in children, classified by the World Health Organization (WHO) as fourth degree tumor the highest malignant grade [13]. Considering the supposed crucial and contradictory functions of ecto-5’-NT in tumor growth, we investigated the role of ecto-5’-NT in MB progression. The enzyme was overexpressed in the D283 human MB cell line in order to evaluate its participation in tumor growth in an *in vivo* nude mice model. Here, we demonstrated that the overexpression of ecto-5’-NT promotes a reduction of tumor growth; interferes with Ki67, CD31 and caspase-3 immunolabeling; and promotes an increase in differentiated tumor cells. In addition, we showed that the expression of A1 adenosine receptor was enhanced, suggesting the participation of adenosine signaling in MB tumor progression.

## Materials and Methods

### Cell culture

Daoy (representative of a human primary MB) and D283 (representative of a secondary or metastatic human MB) cell lines (generated by American Type Culture Collection, ATCC) were kindly donated by the Laboratório de Pesquisa em Câncer Infantil of Hospital de Clínicas de Porto Alegre in Rio Grande do Sul, Brazil. The cells were cultured in low glucose Dulbecco´s Modified Eagle´s Medium (DMEM), supplemented with 10% fetal bovine serum (FBS) and 0.5 U/mL penicillin/streptomycin antibiotics. The cells were kept at 37°C in an incubator with a minimum relative humidity of 95% and 5% of CO_2_.

### Transient D283 cell line transfection

The D283 MB cell line was seeded in culture flasks, and after reaching 80% confluence, the cells were transfected with Lipofectamine^®^ 2000 Transfection Reagent (Invitrogen *by* Life Technologies—Carlsbad, CA, United States). We used 1 μg of pcDNA3.1/V5-His plasmid as a transfection control (D283 empty vector—D283ev) or pcDNA3.1/V5-His containing the human ecto-5’-NT gene sequence (D283hCD73) and incubated for 4 h [14]. Then, the cells were kept in DMEM with 10% FBS for 24 h. The transfected cells were selected based on their resistance to the antibiotic G418 (1.0 mg/mL). The functionality of the pcDNA3.1/V5-His plasmid (D283ev) or pcDNA3.1/V5-His encoding ecto-5’-NT (D283hCD73) sequences was confirmed by evaluating the ecto-5’-NT mRNA and protein expression and ecto-5’-NT activity.

### RT-PCR and Real-Time PCR

Total RNA from MB cell lines was isolated with Trizol^®^ Reagent (Invitrogen *by* Life Technologies—Carlsbad, CA, United States). The cDNA was synthesized with RevertAid reverse transcriptase (Fermentas *by* Life Technologies—Carlsbad, CA, United States) from 3 μg of total RNA in a final volume of 20 μL in the presence of oligo dT primer. The following PCR reaction was performed in a total volume of 25 μL, which included 1 μL of each forward and reverse primers to ecto-5’-NT coding sequences (S1 Table) and 1 μL of Taq DNA polymerase enzyme (Fermentas *by* Life Technologies—Carlsbad, CA, United States). The PCR products were analyzed on a 2.0% agarose gel containing ethidium bromide and visualized under ultraviolet light. The plasmid containing the sequences for ecto-5’-NT was used as a positive expression control for this enzyme. A negative control reaction was performed by substituting the templates for DNase/RNase-free distilled water.

Real-time PCR analysis was performed in the ABI Step One Plus Instrument using the SYBR Green amplification System (Applied Biosystems, Foster City, CA). Each reaction was performed with 0.25 μL of each forward and reverse primer (10 μM) (S1 Table). Because the efficiency of all of the reactions was >95%, the ΔΔCt parameter was used to determine the relative expression levels, using GAPDH gene expression as an endogenous control for normalization.

### Flow Cytometry

#### Ecto-5’-NT

For flow cytometry analysis, one million cells were washed twice with phosphate buffered saline (PBS) plus 1% fetal calf serum (FCS) and centrifuged. The pellets were resuspended and incubated for 1 h with purified mouse anti-human CD73 antibody (1:10, BD Pharmingen TM) for 1 h at 4°C. Next, all of the samples were washed and incubated for 1 h with Alexa Fluor 555 rabbit anti-mouse (1:100) at room temperature. Then, the labeled cells were washed with PBS and immediately analyzed by flow cytometry (Beckman Coulter Fc500). Fifty thousand events in the cell gate were collected and further analyzed using the FlowJo^®^ 7.6.3 software.

#### Active caspase-3 measurement

To evaluate caspase 3 immunolabeling, MB cell lines were seeded and cultivated until 70% confluence. For sequencing, the cells were washed twice and then resuspended in BD Cytofix/Cytoperm^™^ solution at a concentration of 3 × 10^5^ cells per 150 μL and incubated for 20 min at 4°C. Afterward, the cells were washed twice with BD Perm/Wash^™^ buffer (1×) at room temperature. Finally, the cells were incubated in BD Perm/Wash^™^ buffer (1×) containing an antibody against active caspase-3 for 30 min at room temperature in the dark and then analyzed by flow cytometry (FACS Caliber, BD Biosciences, San Jose, CA, USA). The data were analyzed using FlowJo^®^ software (USA).

### Ecto-5’-NT enzymatic assay and protein determination

For the determination of AMP hydrolysis, MB tumor cells were incubated for 10 min with 2 mM AMP diluted in incubation buffer, followed by the determination of the released inorganic phosphate (Pi) [9]. The specific activity was expressed as nmol Pi released/min/mg of protein (nmol Pi/min/mg). The protein concentration was determined by the Coomassie blue method using bovine serum albumin (BSA) as a standard. To determine the effect of APCP on AMPase activity, the cell lines were pre-incubated for 10 min with different APCP concentrations (1, 5, 10, 20 and 50 μM) diluted in incubation buffer. At sequencing, MB tumor cells were incubated with 2 mM AMP diluted in incubation buffer plus the respective APCP concentrations, and the protocols were performed as described above.

### Cell counting

Cells were washed twice with PBS, and 100 μL of 0.25% trypsin/EDTA solution was added to detach the cells prior to counting them with a hemocytometer. According to specific protocols, the cells were prepared as follows: to evaluate the transfection effects on cell proliferation, 10^4^ cells per well were seeded in 24-well plates and allowed to grow for 5 days. A cell count was performed daily. To determine the APCP effect of cell line proliferation, the cells were seeded, and when they reached 60% confluence, the cells were treated. After 24 and 48 h of treatment, cell counting was performed.

### Animal care and tumor implant

All of the experiments were performed in accordance with Ethical Principles in Animal Research as adopted by the Brazilian College Laboratory of Animal Experimentation (COBEA) with prior approval by the Internal Animal Care and Use the Ethical Committee of the Instituto de Química at Universidade de São Paulo (Protocol number 15/2013, 09/20/2010). The animals were maintained in the Animal Facility of the Instituto de Química at the Universidade de São Paulo in Brazil. Balb/c immunodeficient (nude) mice that were 6 to 14 weeks old were used to perform the dorsal subcutaneous xenograph tumor implant model. One million cells were diluted in 200 μL of DMEM and supplemented with 10% FBS and 200 μL of BD Matrigel^™^ Basement Membrane Matrix (BD PharmingenTM—San Jose, CA) and then injected into the dorsal flank of nude mice. The animal groups were set up as follows: control (injected with 200 μL of DMEM with 10% FBS plus 200 μL of Matrigel without cells), Daoy (injected with wild type cells expressing ecto-5’-NT), D283ev (injected with cells with transfection control—empty vector) and D283hCD73 (injected with cells overexpressing ecto-5’-NT). Animals were monitored weekly regarding their weight and tumor growth. Tumor growth measurements performed with a pachymeter twice a week were based on the biggest and smallest diameter of the tumor mass. The tumor size was determined with the equation *TS = (π/6) × A × B* ^*2*^ (*TS*—tumor size; *A*—greater tumor mass diameter; *B*—smaller tumor mass diameter). After the first tumor of each group reached approximately 18 mm at the largest diameter of the tumor mass, all the animals in the group were euthanized by cervical dislocation. The tumors were excised, and their weights and sizes were determined. Tumor masses were fixed with formalin for posterior analysis. To maintain the physical integrity of animals after tumor development, pain signals were monitored, as well as weight loss and apathy. In the case of any of these symptoms, the animals were euthanized. The observation of the animals did not showed that any of them became severely ill or injured during the course of the study. These parameters were established in accordance with the Tumor Policy for Mice and Rats, as described by Boston University Research Compliance—Research Committees, which is accepted by IACUC (Institutional Animal Care and Use Committee), and analyzed and approved by the local Committee as described above.

### In vivo nude mice imaging

Nude mice received an injection containing IRDYE^®^ 800 CW PEG Contrast Agent (1.5 nmol) into the tail artery. After 24 h, the animals were anesthetized with flank injections of ketamine and xylazine (6.67 μL/g) and imaged using the Odyssey^®^CLx Infrared Imaging System plus MousePOD *in vivo* Imaging Accessory. The images were analyzed by the Odyssey^®^CLx software.

### Histopathology and immunohistochemical staining

For histopathological analysis, samples of MB tumors were formalin-fixed, paraffin-embedded and sectioned at a 5-μm thickness. For each sample, a section was stained using standard hematoxylin and eosin (H&E) protocols. Other sections of these samples were used for immunohistochemistry protocols. For this, sections of 5 μm thickness were processed using antigenic Tris/EDTA recuperation at pH 9.0 and high temperature and blocked with methanol (3%) and FBS (5%), followed by overnight incubation at 4°C with the following specific antibodies: anti-human CD31, anti-synaptophysin, anti-enolase, anti-Ki67 (Cell Marque, Rocklin, CA), anti-human CD73 (Santa Cruz Biotechnology, INC.—Dallas, Texas) and polyclonal rabbit antibody against active Caspase 3 (Abcam, Cambridge, MA). Next, tissue sections were incubated with the Reveal Complement secondary antibody (Spring Bioscience). The H&E and Immunoblotting slides were analyzed by a pathologist in a blind manner. Each positive endothelial cell cluster of immunoreactivity in contact with the selected field was considered an individual vessel. For all of the antigens analyzed here, the expression and localization were considered positive only when the cells were clearly immunoreactive. Pathological evaluations were performed for ten randomly chosen fields (× 200) per tumor, using an Olympus BH-2 microscope. Blind quantitative analyses of five randomly chosen images of each tumor sample were performed for Ki67 and CD31 immunolabeling, using ImageJ as the image analysis program.

### Statistical Analysis

Data were expressed as the mean value ± S.D. of at least three independent experiments and were subjected to a One-way analysis of variance (ANOVA) followed by Tukey’s post-hoc tests. The differences between the mean values were considered significant when *p<0*.*05*.

## Results

### Analysis of ecto-5’-NT expression and activity in MB transfected cells

**Fig 1:**
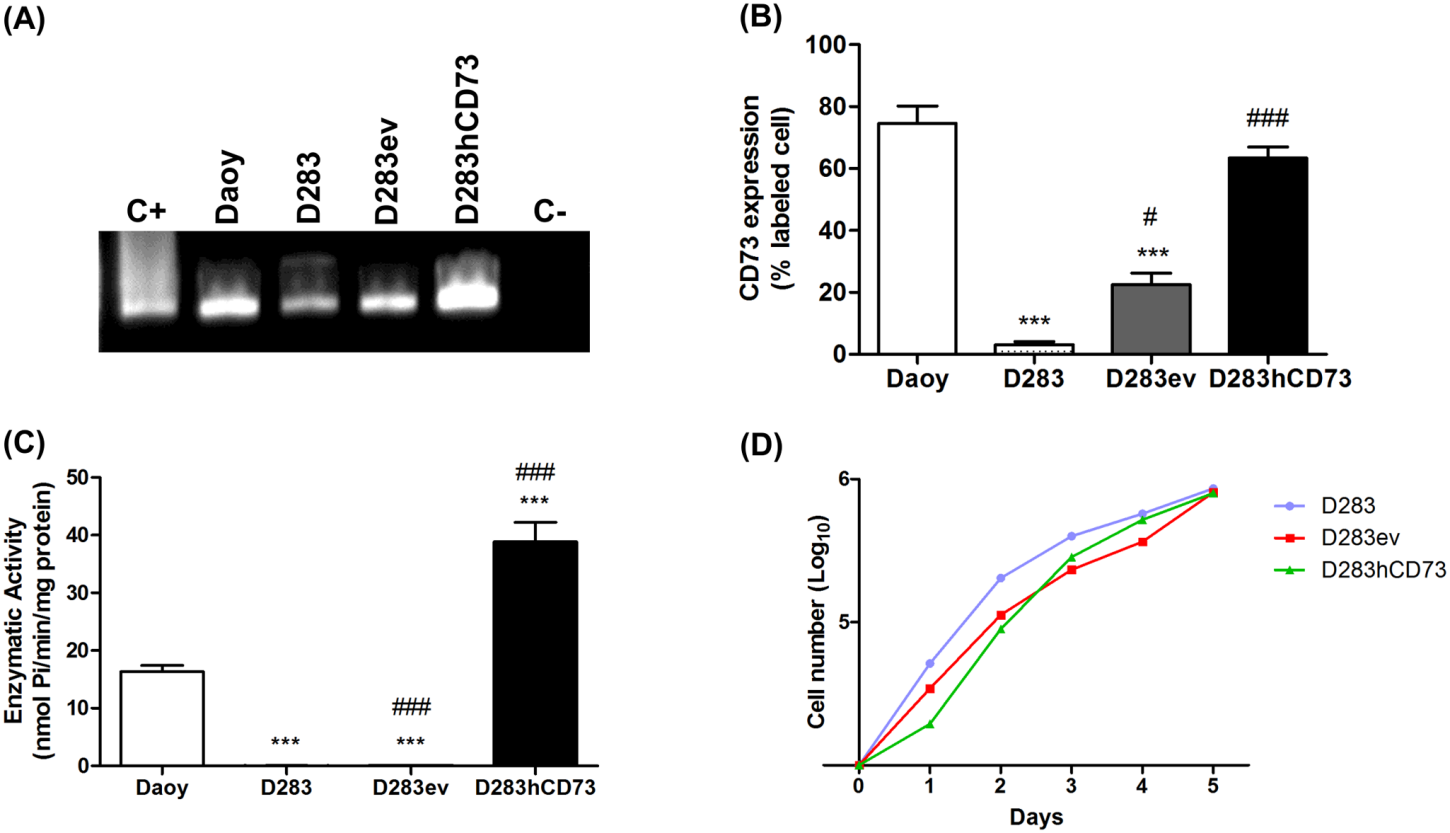
Ecto-5’-NT expression and activity following transfection of the D283 MB cell line. Ecto-5’-NT expression was determined by **(A)** RT-PCR analysis, **(B)** flow cytometry and **(C)** enzymatic activity as described in the Materials and Methods. **(D)** Cell proliferation indices for each cell line were obtained during five days of culture. (*) p < 0.05; (**) p < 0.01; and (***) p < 0.001 indicate significant differences compared to the Daoy cell line, and (#) p < 0.05; (##) p < 0.01; and (###) p < 0.001 indicate significant differences compared to the D283 cell line.

The expression of ecto-5’-NT in transfected cells (D283hCD73), as well as in Daoy, D283 and D283ev (empty vector control), was detected on the gene and protein expression levels (Fig 1). The Daoy MB cell line was used as a positive control for ecto-5’-NT expression and D283 as a cell line expressing low levels of this enzyme [9]. Gene expression analysis revealed that the D283hCD73 MB cell ecto-5’-NT expression levels are low in D283ev cells (Fig 1A), in agreement with the flow cytometry results (Fig 1B). Daoy and D283hCD73 cells were positive for anti-ecto-5’-NT immunofluorescence staining, with no significant differences (Fig 1B). The obtained expression profiles were in agreement with the enzymatic activity levels, revealing the enhanced functionality of ecto-5’-NT in the D283hCD73 cell line in converting AMP into adenosine (Fig 1C). Ecto-5’-NT overexpression and the transfection process *per se* did not affect the *in vitro* proliferation of MB cells (Fig 1D).

### Evaluation of AMPase activity and cell proliferation after ecto-5’-NT inhibition

**Fig 2:**
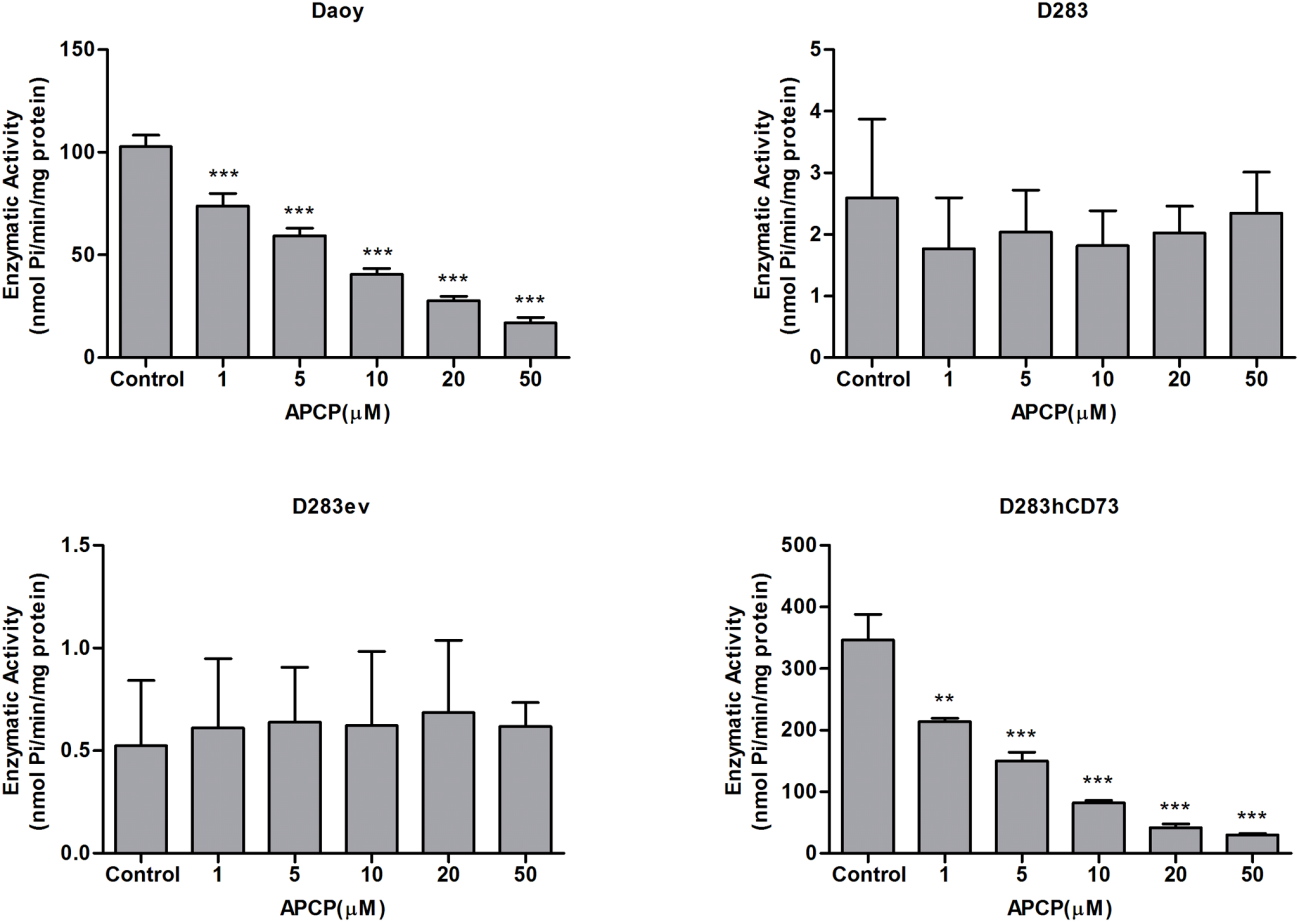
Effect of APCP on AMPase activity in human MB cell lines. After reaching confluence, MB cell lines were pre-incubated for 10 min with APCP at the following concentrations: 1, 5, 10, 20 and 50 μM. At sequencing, AMP was added as a substrate at 2 mM for all cell lines. For Daoy and D283hCD73, the cells were incubated for 10 min and for D283 and D283ev, 30 min. The control did not receive APCP at any time. Specific activities were expressed as nmol/Pi/mg of protein. (*) p < 0.05; (**) p < 0.01; and (***) p < 0.001 indicate significant differences compared to the control of each respective cell line.

**Fig 3:**
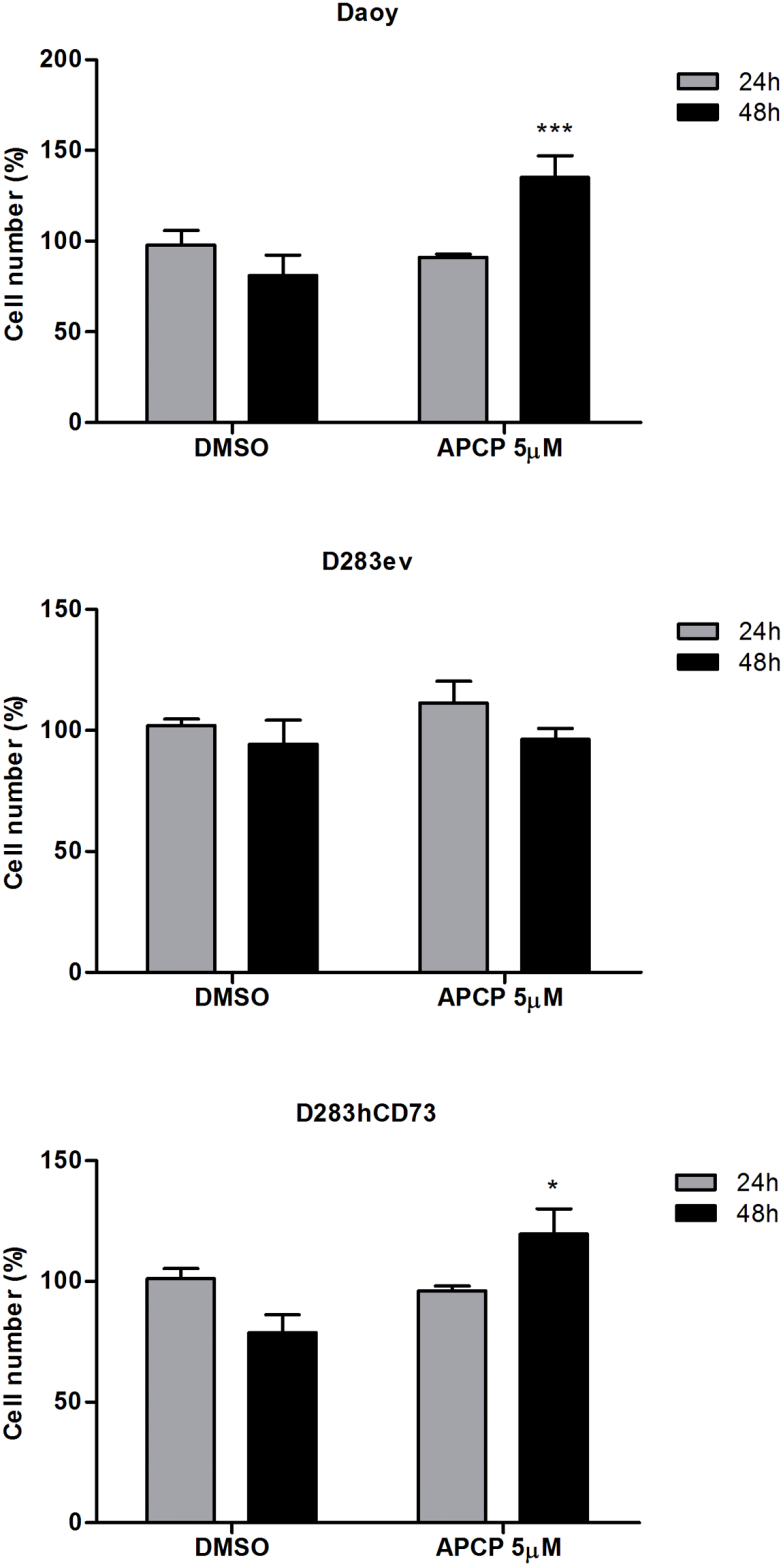
Effect of APCP on MB cell proliferation. At 60% confluence, the cells were treated with 5 μM APCP for 24 and 48 h, and cell counting was performed as described in the Materials and Methods. Controls were considered 100%. The data were analyzed by a Student t-test, and (*) p < 0.05 and (***) p < 0.001 indicate significant differences compared to the control of each respective cell line.

First, we determined the optimal concentration of APCP, a specific ecto-5’-NT inhibitor that inhibits the enzymatic activity in the cell lines Daoy, D283, D283ev and D283hCD73 (Fig 2). We could observe that D283 and D283ev demonstrated a very low enzymatic activity, corresponding to the low ecto-5’-NT expression that is presented by these cell lines. Thus, it is possible to infer that APCP did not alter this behavior. Daoy and D283hCD73 presented a prominent AMP hydrolysis, and APCP efficiently inhibited ecto-5’-NT activity at all of the tested concentrations (Fig 2). During sequencing, to determine the influence of ecto-5’-NT on MB cell proliferation, we used 5 μM APCP for 24 and 48 h. After 48 h, APCP stimulated the cell proliferation in the Daoy (54%) and D283hCD73 (41%) cell lines (Fig 3). These data suggest that the inhibition of ecto-5’-NT promotes cell proliferation in MB cell lines that express this enzyme. No difference was observed in D283ev cell proliferation at any time evaluated. D283 was not tested because it does did show a significant difference compared to D283ev, the transfection control cell line.

### The effect of ecto-5’-NT overexpression on the MB xenograph in vivo model

**Fig 4:**
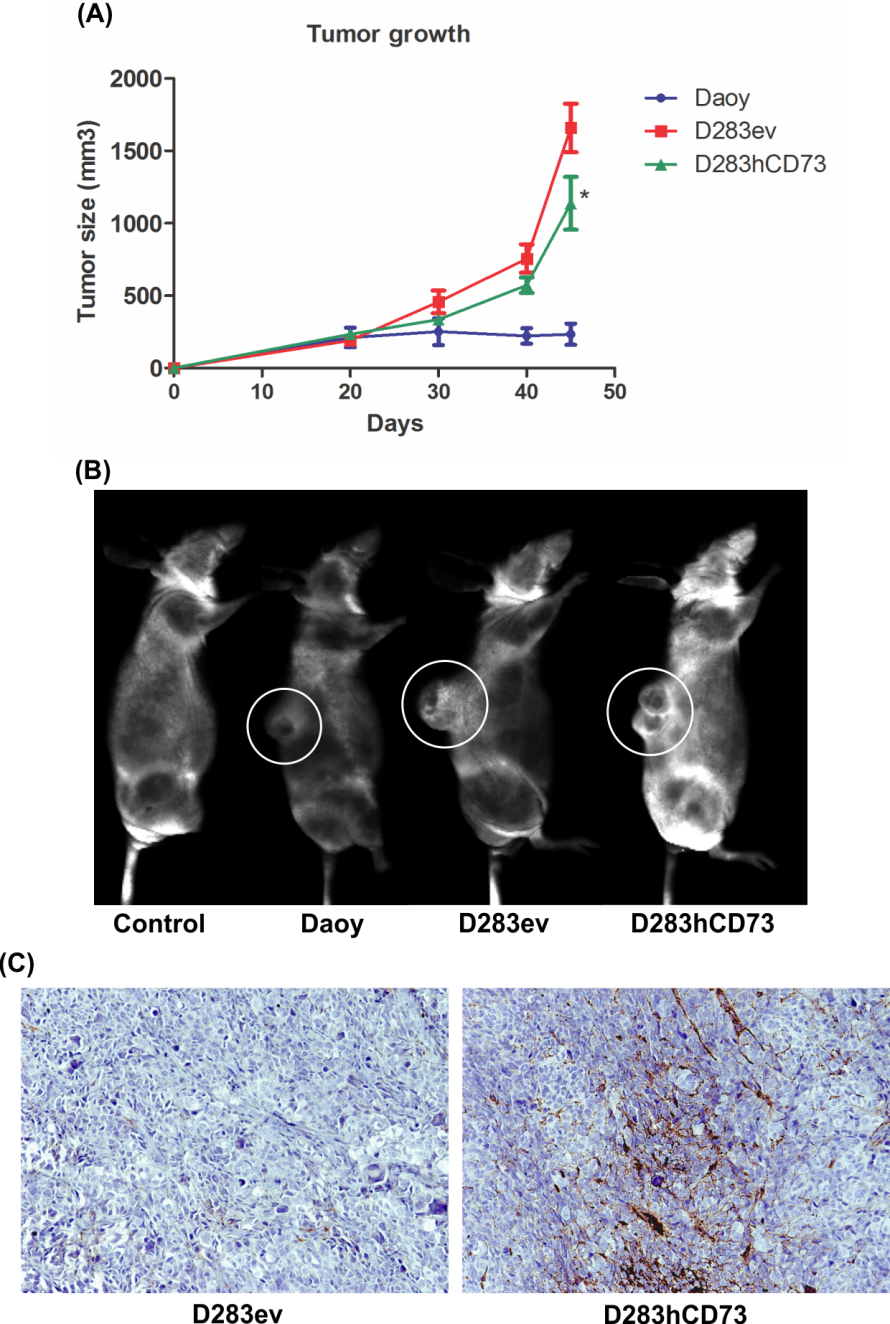
Ecto-5’-NT expression in D283 decreases the growth of tumor cells. To determine human MB tumor growth in nude mice, equal amounts of Daoy, D283ev and D283hCD73 cells (1 × 10^6^ cells) were implanted by subcutaneous injection into the dorsal region of nude mice. During tumor growth, the following data were obtained: **(A)** Measurements of tumor mass, which determine tumor growth (mm^3^). **(B)** Prior to euthanasia, nude mice were injected with the IRDYE^®^ 800 CW PEG Contrast Agent, and images were captured in the Odyssey^®^CLx Infrared Imaging System plus MousePOD *in vivo* Imaging Accessory. Thus, the location and size of the tumor could be qualitatively measured *in vivo*. The white circle highlights the tumor mass in each animal that was examined. **(C)** Detection of ecto-5’-NT immunoreactivity in D283ev and D283hCD73 tumors. The values represent the mean values ± SD (n = 10) for each analyzed group, where (*) p < 0.05.

The effects of ecto-5’-NT overexpression on *in vivo* MB growth were assessed after the subcutaneous injection of Daoy, D283ev or D283hCD73 cells in a nude mouse xenograph model. No changes in the body weight of the animals were noted during tumor growth (S1 Fig). Measurements of the maximal and minimal diameters showed that the D283ev cell line produced larger tumors compared to the other transplanted tumor type (Fig 4A and 4B and S2 Fig). The Daoy cell line generated the lowest mass within the studied tumors (S2 Fig). An important result that was obtained in this work was the reduction of the tumor growth presented by the D283hCD73 cell line (Fig 4A and 4B and S2 Fig), suggesting that the overexpression and increased activity of ecto-5’-NT favor the reduction of MB tumor growth in this *in vivo* model. This observation is supported by the final tumor size and weight (S1 Fig). Both results demonstrate the evident effect of ecto-5’-NT overexpression on promoting tumor reduction compared to the D283ev control. At the same time of tumor growth, Daoy cells with elevated endogenous ecto-5’-NT expression generated a smaller tumor mass. In addition, the immunoreactivity levels for ecto-5’-NT confirmed the differences between ecto-5’-NT expression rates, in accordance with the produced tumor mass, where the tumors that originated by D283hCD73 showed the most prominent reactivity for ecto-5’-NT expression, while the D283ev tumors were negative (Fig 4C).

### The characterization of xenograph human MB tumors and histopathological analysis

**Fig 5:**
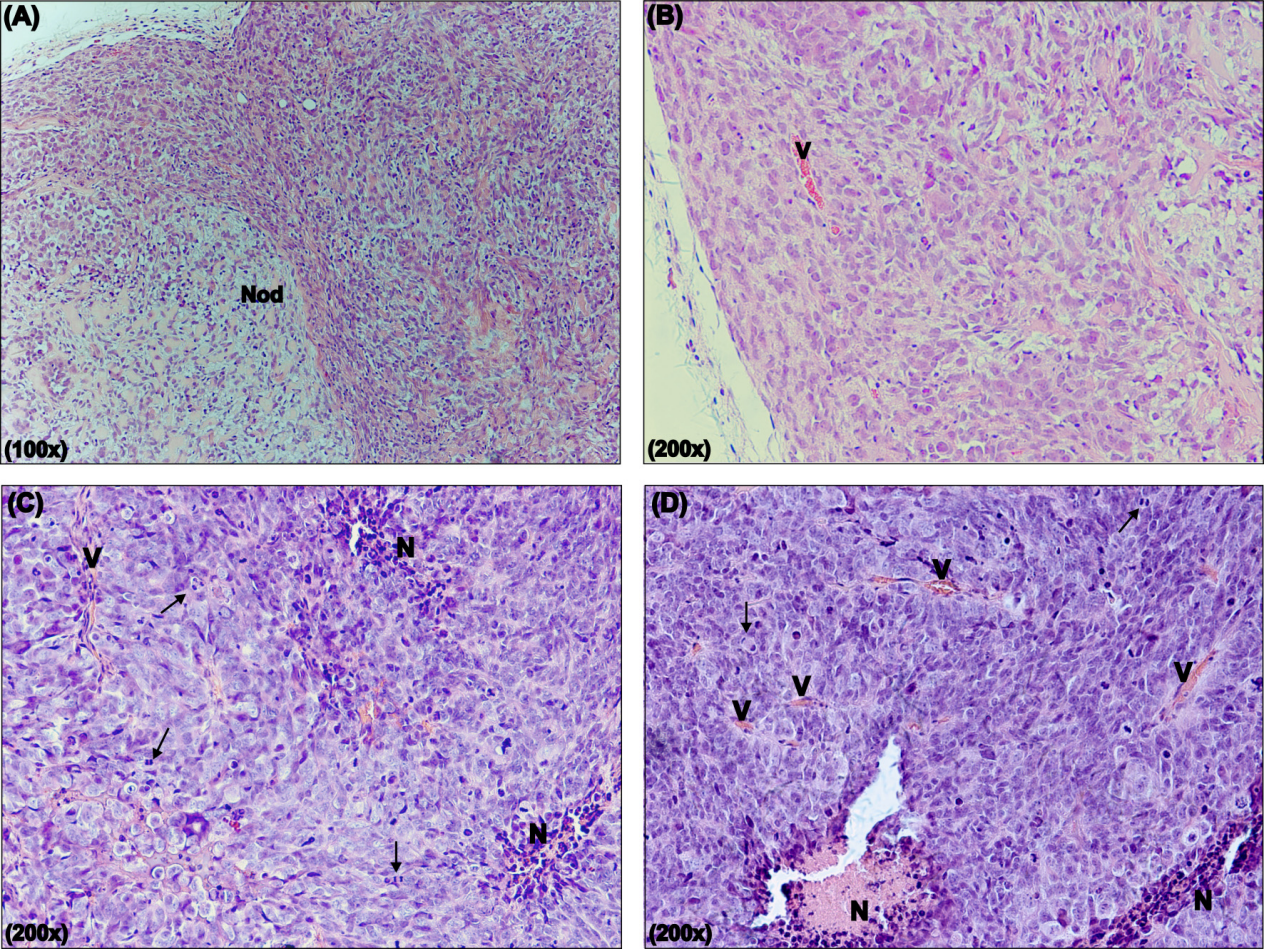
Histopathological analysis of human MB implanted tumors. The implanted tumors were excised, fixed and destined to posterior analysis as described in the Materials and Methods. Representative H&E sections of MB tumors **(A, B)** Daoy, **(C)** D283ev and **(D)** D283hCD73 demonstrate the histopathological characteristics of human MB: the presence of nodularity (Nod), intratumoral vascularization (V), necrotic areas (N) and the presence of mitosis are indicated by arrows. The results of additional analyses are detailed in Table 1. The images were obtained using an inverted fluorescence microscope (Nikon Eclipse TE 300).

The pathological analysis of the implanted tumors demonstrated that all of the samples presented characteristics of MB (Fig 5; Table 1). The histopathological analysis showed that Daoy-implanted tumors presented areas of nodularity, accompanied by accentuated cellular atypia and moderate cellularity. Additionally, these tumors presented a low mitotic index and poor vascularization of the tumor tissue. On the other hand, D283ev and D283hCD73-transplanted animals showed augmented cellular atypia and cellularity with extensive necrosis areas. The mitotic index that was presented by D283hCD73 MB tumor samples was lower than that of D283ev (Table 1). The reduced proliferation and size of the tumors generated by this cell line correlate with the enhanced expression of ecto-5’-NT.

**Table 1:** Histopathological characteristics of implanted MB.

	Cellularity	Atypia	Necrosis	Mitotic index
**Daoy (n = 6)**	Moderate (4/6)	Accentuated (3/6)	Absent (6/6)	14.5
**D283ev (n = 10)**	Accentuated (10/10)	Accentuated (10/10)	Present (10/10)	79.8
**D283hCD3 (n = 9)**	Accentuated (8/9)	Accentuated (8/9)	Present (8/9)	64.7

The slides of H&E were analyzed by a pathologist in a blinded manner. Analysis was performed using ten randomly chosen fields (x 200) per tumor (Olympus BH-2 microscope).

**Fig 6:**
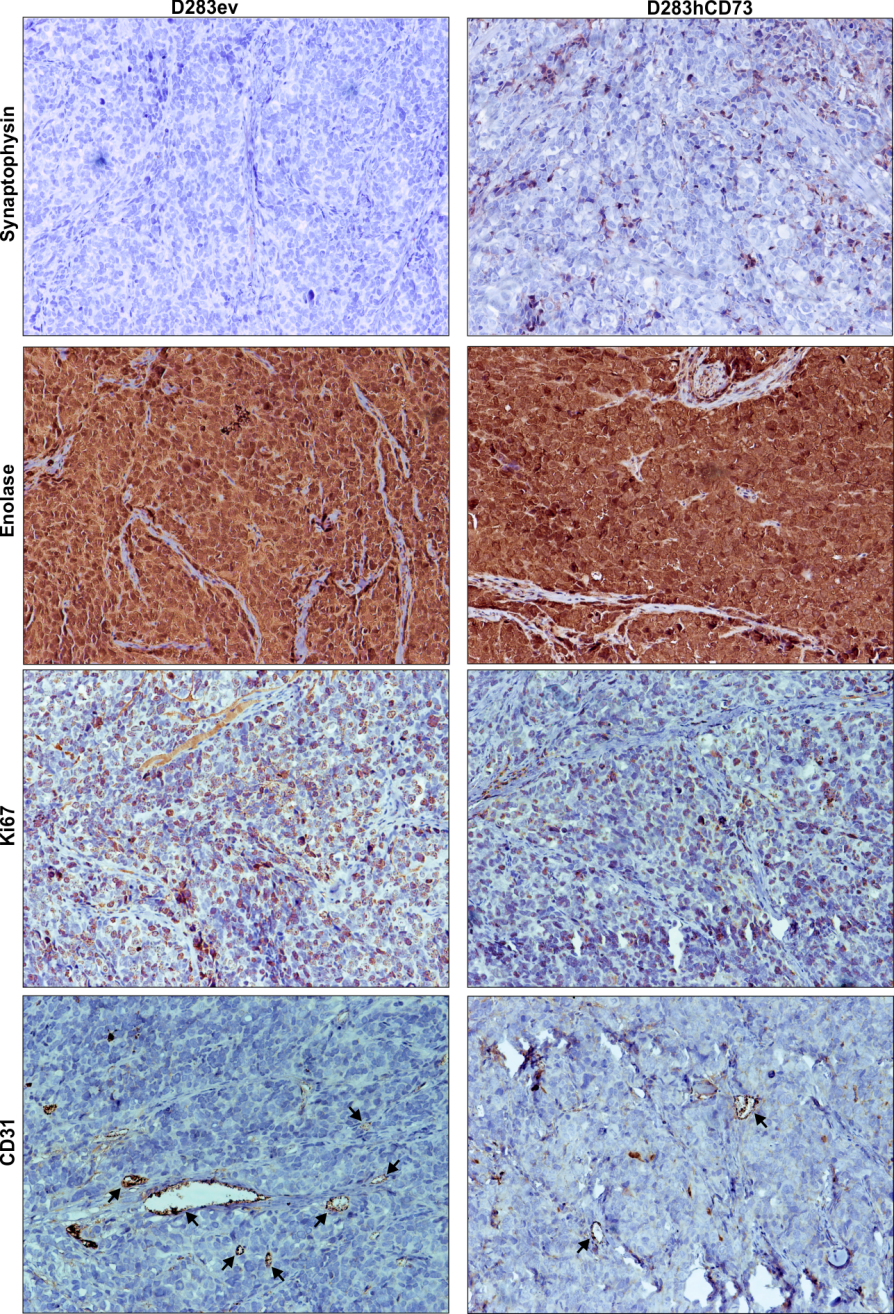
Immunohistochemical characterization of engrafted D283ev and D283hCD73 human MB tumors. Immunohistochemical labeling was performed as described in the Materials and Methods. Anti-synaptophysin and enolase immunostaining were used to evaluate whether the tumor samples were representative of human MB. The proliferation profile was determined using the anti-human Ki67 antibody. Tissue vascularization was visualized with an anti-human CD31 antibody. Labeling is indicated by arrows. Additional analyses are described in Table 2 and S3 Fig.

Implanted tumors represented human MB, as revealed by immunohistochemical staining for the specific markers enolase and synaptophysin. In addition, Ki67 and CD31 characterize this tumor type (Fig 6 and Table 2). Intensive and diffuse reactivity to enolase was present in D283ev and D283hCD73 tumor samples. Furthermore, D283ev tumor samples did not present immunoreactivity for synaptophysin, a marker of differentiated cells. The absence of this marker in D283ev demonstrates that this tumor was highly undifferentiated. On the other hand, synaptophysin expression, as observed in D283hCD73 samples, revealed an expressive population of differentiated cells, characterizing it as less aggressive. In agreement, D283hCD73-originated tumors showed less staining for the proliferation antigen Ki67 than did D283ev tumors (Fig 6, Table 2 and S3 Fig). The immunoreactivity for CD31, a vessel marker, was less evident in D283hCD73 tumor samples, indicating that this tumor is also less vascularized than the tumor originated by D283ev (Fig 6, Table 2 and S3 Fig).

**Table 2:** Immunohistochemical analysis of implanted MB.

	Enolase	Synaptophysin	Ki67	CD31	Ecto-5’-NT
**D283ev (n = 3)**	Positive diffuse (3/3)	Absent (3/3)	90% (3/3)	Strong positivity (3/3)	Absent (3/3)
**D283hCD7 (n = 3)**	Positive diffuse (3/3)	Positive focal (3/3)	75% (3/3)	Weak positivity (3/3)	Positive focal (3/3)

Immunohistochemistry slides were analyzed by a pathologist in a blinded manner. Analysis were performed using ten randomly chosen fields (x200) per tumor (Olympus BH-2 microscope). Ki67 positive cells were quantified by counting labeled cells in each field.

**Fig 7:**
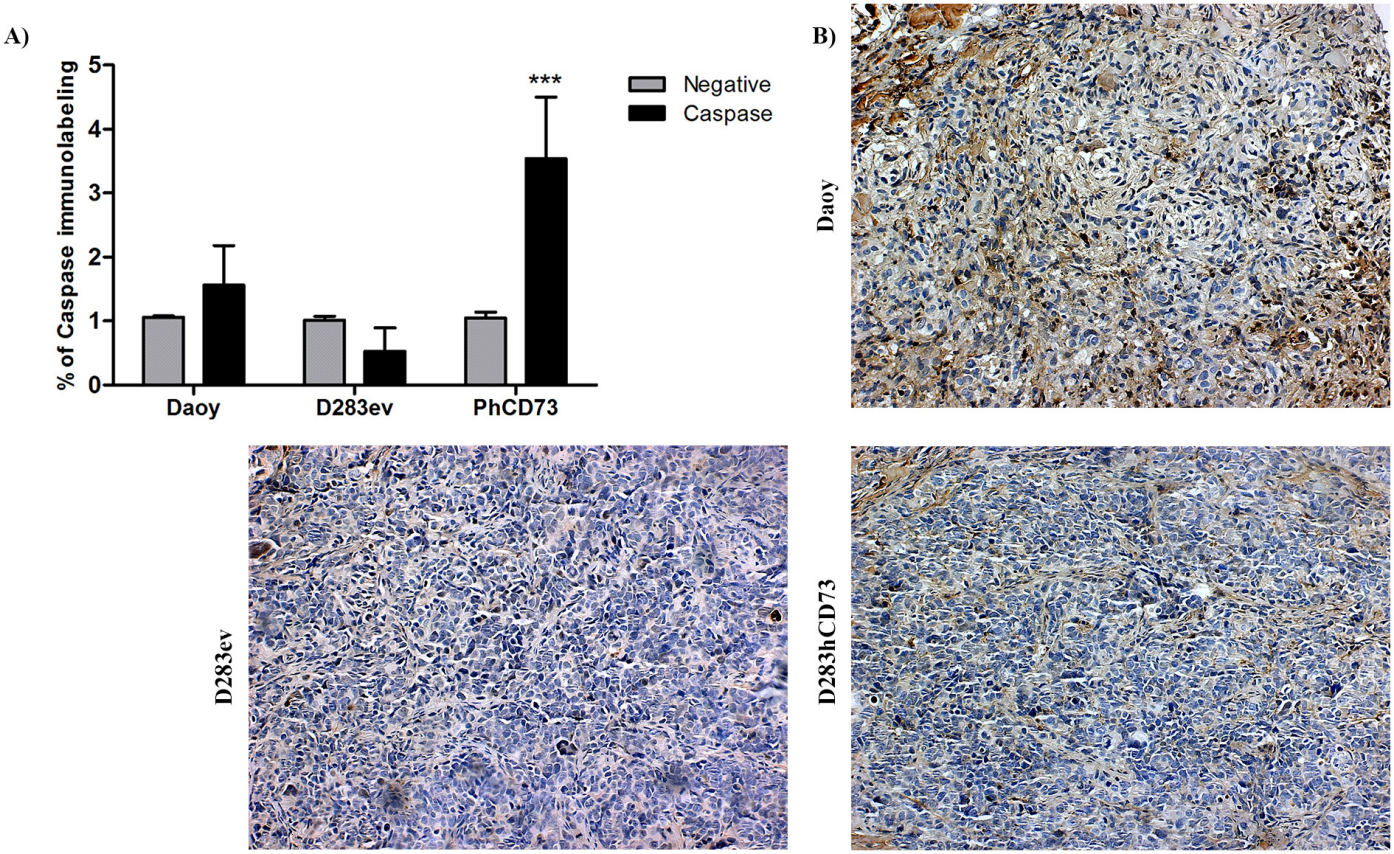
Evaluation of immunolabeling of active Caspase-3. Caspase-3 immunolabeling was evaluated in MB tumor cells lines by flow cytometry (A) and tumor samples by immunohistochemical staining (B) as described in the Materials and Methods. (***) p < 0.001 indicates a statistically relevant difference in relation to negative cells. The images were obtained using an inverted fluorescence microscope (Carl Zeiss-Imager M2 microscope) at 20× magnification.

In addition, we evaluated the immunolabeling to active caspase-3 in MB cell lines by flow cytometry and in MB tumor samples by immunohistochemistry. We observed that Daoy and D283hCD73 cell lines presented the highest staining for active caspase-3, where D283hCD73 showed a significant difference (Fig 7A). Although the values represented the basal levels of caspase-3 expression, the significant difference presented by D283hCD73 in relation to D283ev reflects elevated apoptosis levels and might be related to the reduced tumor growth. In agreement, with immunohistochemistry labeling for active caspase-3, D283hCD73 and Daoy tumor samples presented the prominent expression of this protein in relation to D283ev (Fig 7B). Given these data, we suggest that the expression of ecto-5’-NT by MB tumor cells can promote alterations in basal apoptosis levels, favoring the reduction of the tumor throughout its growth.

### P1 receptor gene expression in human MB cell lines

**Fig 8:**
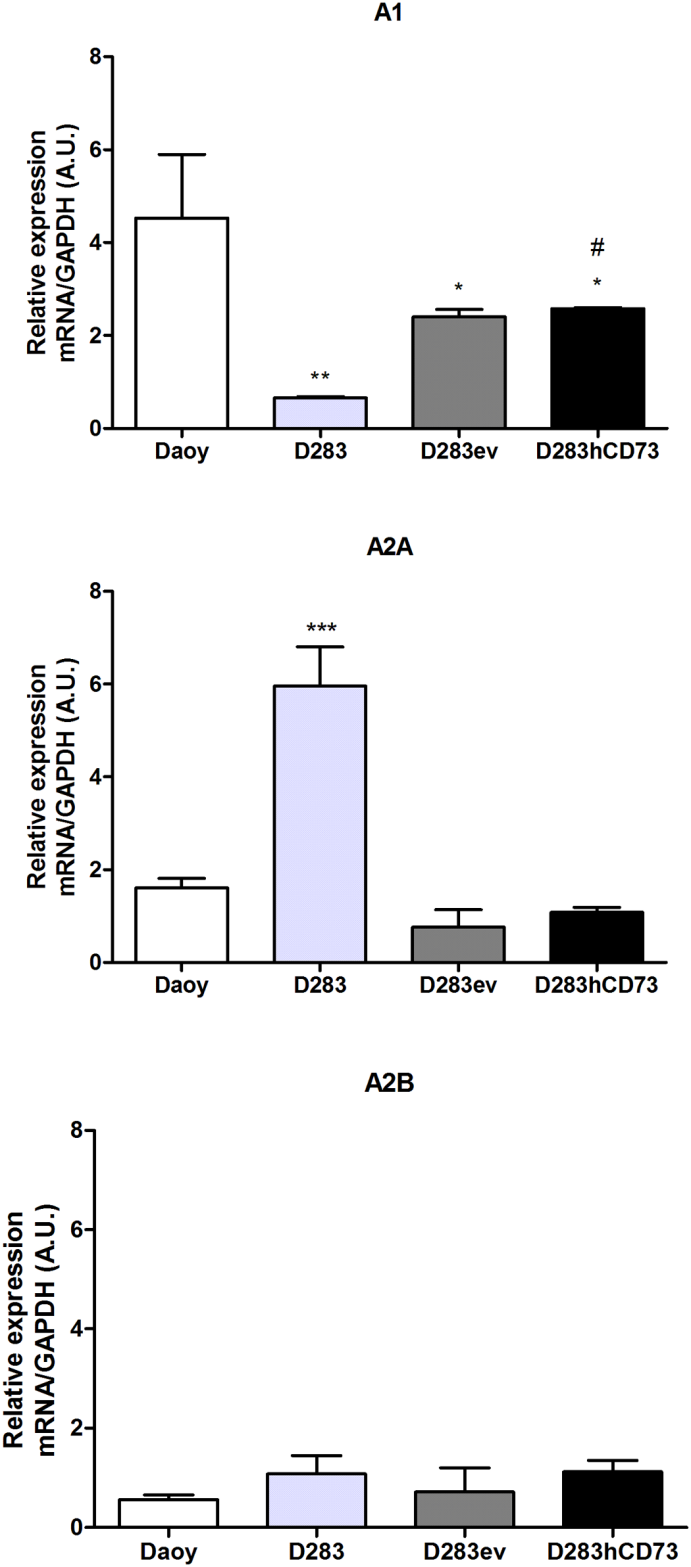
P1 adenosine receptor expression in human MB lines. The relative expression levels of A_1_, A_2A_, A_2B_ and A_3_ in Daoy, D283, D283ev and D283hCD73 MB cell lines were assessed by real-time PCR. Endogenous GAPDH expression was used to normalize the adenosine expression levels. * p < 0.05, ** p < 0.01 and *** p < 0.001 indicate a significant difference between the analyzed samples and the Daoy cell line; # p < 0.05 corresponds to differences between the analyzed samples and the D283 cell line.

The expression levels of the A_1_ adenosine receptor were higher in Daoy cells than in the D283 cell line (Fig 8). D283ev and D283hCD73 cells also revealed diminished A_1_ receptor expression levels compared to Daoy cells. On the other hand, D283 cells showed the most prominent expression of the A_2A_ adenosine receptor. All of the other cell lines presented low expression rates of A_2B_ adenosine receptors. A_3_ adenosine receptor expression could not be detected in these cell lines. The altered expression of P1 receptors as observed in transfected cells might be related to the transfection process because A_1_ and A_2A_ expression were not different in D283ev and D283hCD73 cells.

## Discussion

To investigate the role of ecto-5’-NT in MB tumor progression, we overexpressed this enzyme in D283 MB cells, generating novel tools for investigating the participation of this enzyme in the process: D283ev was used as a transfection control, and D283hCD73 were the cells that received the ecto-5’-NT human sequence. First, we performed experiments to evaluate the efficiency of transfection. The obtained results showed that D283hCD73 expressed higher levels of ecto-5’-NT mRNA and translated protein (Fig 1A and 1B). Moreover, this transfected cell line showed higher AMPase activity than did D283 wild type and D283ev cells (Fig 1C), confirming that the ecto-5’-NT transfection was successful. At sequencing, we could see that APCP *in vitro* could efficiently inhibit ecto-5’-NT activity (Fig 2A and 2D) and promote cell proliferation in Daoy and D283hCD73 (Fig 3A and 3C).

The most important result was that the inhibition of ecto-5’-NT *in vitro* promotes cell proliferation, while *in vivo*, its overexpression promotes the reduction of tumor growth, demonstrating that the modulation of this enzyme can promote alterations in the MB cell proliferation levels, where its expression and activity can reduce this cell event. Immunoreactivity for ecto-5’-NT presented by D283hCD73 tumor slices confirms its overexpression in the tumor mass that presented reduced tumor growth (Fig 4). The pharmacological modulation of ecto-5’-NT promotes increased activity and a reduction of glioma cell proliferation *in vitro* [15, 16]. Indomethacin, in addition to activating ecto-5’-NT, also promotes the expression of A_3_ adenosine receptor, which induces cell death [16]. In this way, knowing that the main function attributed to ecto-5’-NT is the extracellular production of adenosine [1], which make its function by activation of P1 adenosine receptors, we also determined the expression profile of P1 receptors in human MB cell lines. Daoy, D283ev and D283hCD73 preferentially expressed A_1_, and D283 expressed prominent levels of the A_2A_ adenosine receptor (Fig 8). Of note, the cell transfection process appeared to increase the expression of all of the adenosine receptors in both the D283hCD73 and D283ev cell lines; nevertheless, in our tumor model, only Daoy and D283hCD73 were exposed to high adenosine levels, due to ecto-5’-NT expression. In pathological environments, ecto-5’-NT presents an important function to modulate the action of adenosine in tumor progression [1]. This nucleoside, by sensitizing P1 receptors, may favor tumor growth by stimulating angiogenesis, cell proliferation and immune response suppression [2]. However, adenosine is capable of generating different cellular behaviors depending on the expression profile of P1 receptors and the cell type expressing these receptors. It has been suggested that adenosine promotes tumor cell apoptosis by intrinsic [17] and extrinsic pathways [18]. Adenosine is taken up by cells into the intracellular medium, where it activates AMP-activated protein kinase (AMPK) and thus promotes apoptosis by caspase-3/-8, as shown for human hepatoma cells [17]. Saito and co-workers (2010) have shown that adenosine suppresses the growth of CW2 human colonic tumor cells by inducing apoptosis, which is mediated by A_1_ receptors [19]. The RCR-1 astrocytoma cell line contains high levels of extracellular adenosine, promoting apoptosis mediated by A_1_ receptor-stimulated caspase-9/-3 activation [18]. In addition, the selective agonism of the A_1_ adenosine receptor reduced cell proliferation in different tumor human cell lines [20]. Given these data, and knowing that the overexpression of ecto-5’-NT promotes an increase in active caspase-3 immunolabeling in MB cell lines (Fig 7), we suggest that this enzyme can induce apoptosis in MB cell lines to activate the A_1_ adenosine receptor to produce adenosine in the extracellular medium.

In addition, D283hCD73 generated a tumor with more differentiated cells, as demonstrated by the enhanced synaptophysin immunoreactivity (Fig 6). In agreement with the results reported here, the samples of patients with prostate carcinoma revealed less ecto-5’-NT expression in tumor tissues compared to normal differentiated prostatic tissue [21]. Furthermore, enhanced ecto-5’-NT expression levels may be related to good prognosis, as suggested by the increased survival rates of these patients. Furthermore, ovarian cancer patients with good prognosis also presented high tumor differentiation levels and ecto-5’-NT expression [22]. Together, these data suggest that the overexpression of ecto-5’-NT in the D283 MB cell line makes it less aggressive and favors the reduction of tumor growth. In agreement with the increased number of differentiated cells, D283hCD73 showed a lower proliferative index, corroborated by a reduced mitotic index and the lowest frequency of Ki67 labeling, as well increased active caspase-3 immunolabeling, which is indicative of apoptosis. This tumor also presented less CD31 immunoreactivity than did D283ev, indicating decreased tumor mass vascularization, which is associated with reduced tumor growth. Thus, taken together, these results could justify the reduced tumor growth presented by the D283hCD73 MB cell line.

All MB types are considered by the WHO (World Health Organization) to be highly malignant tumors [13]. However, we suggested that MB tumor progression depends on ecto-5’-NT expression levels. Animals that were injected with Daoy MB cells generated a large tumor only after four months of growth (S4 Fig), in contrast to the tumors generated by the D283ev cell line, which achieved their maximum size following 45 days of inoculation. Because the Daoy MB cell line was also able to generate a malignant tumor (S2 Table), the results presented here suggest that ecto-5’-NT expression may not be related to the degree of malignancy but rather to decreased tumor growth, making it slower and thus increasing the survival time. Ecto-5’-NT expression is supposedly regulated by the Wnt/β-catenin canonic pathway, which has been considered a good prognostic marker for MB patients [9, 11]. Thus, although all MB are malignant, these data suggest that the expression of ecto-5’-NT by this type of childhood tumor can be associated with good prognosis, where these tumors present a slower growth and become susceptible to therapy.

In view of the data presented here and the literature review, we suggest that the reduction of the tumor growth after ecto-5’-NT overexpression can be attributed to two possible events: 1) an increase of differentiated cells in the tumor mass generated by D283hCD73 that makes the tumor cells less proliferative and 2) the activation of A_1_ adenosine receptors expressed by D283hCD73 cells, which can promote apoptosis and slow tumor growth.

Finally, ecto-5’-NT positively affects the cancer progression of different types of adult tumors (breast and bladder cancer, melanoma and glioma) [1]. In specific childhood tumors, such as MB, this enzyme is involved with a subtype that presents a profile with good prognosis [9]. Adult tumors can present behaviors different from those of childhood tumors, and the literature emphasizes that a comparison between these behaviors cannot be routinely performed [23, 24]. This factor could also be considered important in explaining why ecto-5’-NT expression in MB is involved in a reduction of tumor growth. In conclusion, these data suggest ecto-5’-NT is an important target for controlling MB progression, suggesting a novel diagnostic tool and a target for therapeutic intervention. Additional studies are necessary to demonstrate the relationship between ecto-5’-NT and P1 adenosine receptors in MB progression.

## Supporting Information

S1 Fig Ecto-5’-NT expression reduces tumor growth in the D283 MB cell line. Following transplantation and maintenance of animals for tumor growth, as stated in Materials & Methods, all animals were euthanized and the following analyses were performed: measurement of animal body weight **(A)**, the final tumor weight **(B)** and tumor size **(C)**. The values represent the mean ± SD with n = 10 for each group analyzed where (*) p < 0.05; (**) p < 0.01; (***) p < 0.001, indicating a statistical difference in relation to the Daoy cell line and (#) p < 0.05; (##) p < 0.01; (###) p < 0.001, indicating a statistical difference in relation to the D283ev cell line.
(DOCX)

S2 Fig Differences in tumor growth after finalization of the *in vivo* experiment. (DOCX)

S3 Fig Quantification of Ki67 and CD31 immunolabeling. Percentages of Ki67- and CD31-positive cells were quantified by immunohistochemistry in MB tumor samples. Five images (x 400) were captured per sample in a random manner using the Carl Zeiss-Imager.M2 microscope and quantified with the ImageJ Software.
(DOCX)

S4 Fig Determination of tumor growth after Daoy cell engraftment. To determine human MB tumor growth in a nude mice *in vivo* model 1 x 10^6^Daoy cells were implanted by subcutaneous injection in the dorsal region of nude mice. During the tumor growth the following data were obtained: **(A)** Measurements of the maximum and minimum diameters of the tumor mass, which determines tumor growth (mm^3^). **(B)** Following finalization of the experiment, all animals were euthanized and the final tumor weight was determined. The values represent mean values ± SD (n = 6) for each analyzed cell group, where (*) p < 0.05 and (***) p<0.001.
(DOCX)

S1 Table Ecto-5’-NT and adenosine receptor primer sequences. (DOCX)

S2 Table Histopathological characteristics of implanted Daoy MB, four months after implantation. (DOCX)
