# Supplementary material for: Ecto-5’-Nucleotidase Overexpression Reduces Tumor Growth in a Xenograph Medulloblastoma Model
Source: PLoS One. 2015 Oct 22;10(10):e0140996. doi: 10.1371/journal.pone.0140996 (PMC4619639; doi:10.1371/journal.pone.0140996)
Supplement: S1 Fig — Following transplantation and maintenance of animals for tumor growth, as stated in Materials & Methods, all animals were euthanized and the following analyses were performed: measurement of animal body weight (A), the final tumor weight (B) and tumor size (C). The values represent the mean ± SD with n = 10 for each group analyzed where (*) p < 0.05; (**) p < 0.01; (***) p < 0.001, indicating a statistical difference in relation to the Daoy cell line and (#) p < 0.05; (##) p < 0.01; (###) p < 0.001, indicating a statistical difference in relation to the D283ev cell line. (DOCX) [file pone.0140996.s001.docx]

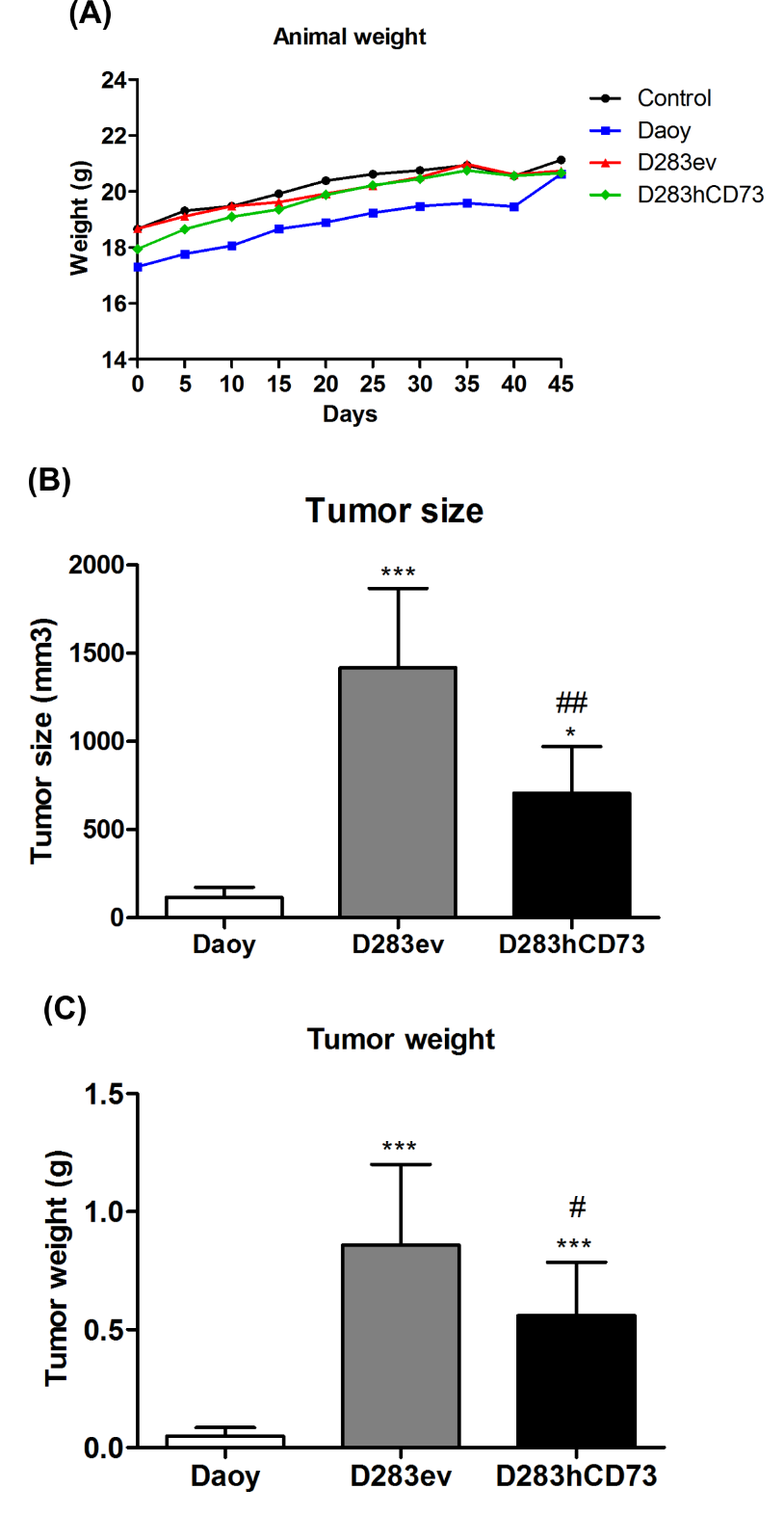


**S1 Fig - Ecto-5’-NT expression reduces tumor growth in the D283 MB cell line.** Following transplantation and maintenance of animals for tumor growth, as stated in Materials & Methods, all animals were euthanized and the following analyses were performed: measurement of animal body weight **(A)**, the final tumor weight **(B)** and tumor size **(C)**. The values represent the mean ± SD with n=10 for each group analyzed where (*) p < 0.05; (**) p < 0.01; (***) p < 0.001, indicating a statistical difference in relation to the Daoy cell line and (#) p < 0.05; (##) p < 0.01; (###) p < 0.001, indicating a statistical difference in relation to the D283ev cell line.
