# Supplementary figures and images for: Ecto-5’-Nucleotidase Overexpression Reduces Tumor Growth in a Xenograph Medulloblastoma Model
Source: PLoS One. 2015 Oct 22;10(10):e0140996. doi: 10.1371/journal.pone.0140996 (PMC4619639; doi:10.1371/journal.pone.0140996)

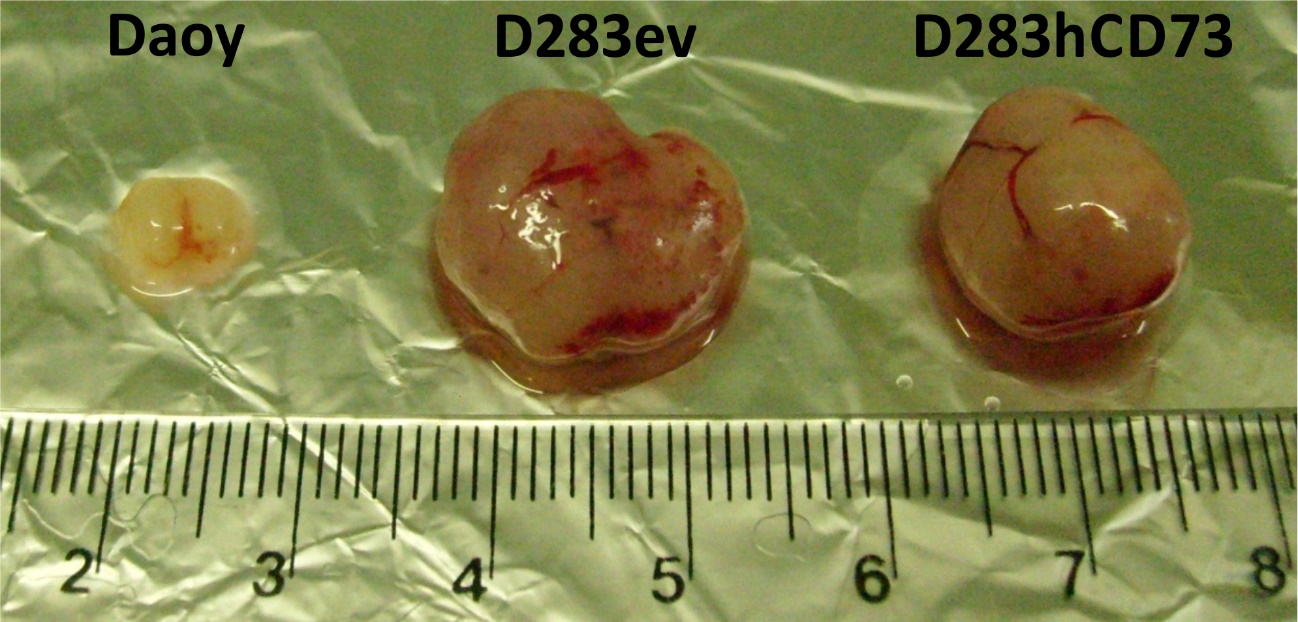


**S2 Fig – Differences in tumor growth after finalization of the *in vivo* experiment.**

Supplement: S2 Fig — (DOCX) [file pone.0140996.s002.docx]
