# Supplementary material for: Ecto-5’-Nucleotidase Overexpression Reduces Tumor Growth in a Xenograph Medulloblastoma Model
Source: PLoS One. 2015 Oct 22;10(10):e0140996. doi: 10.1371/journal.pone.0140996 (PMC4619639; doi:10.1371/journal.pone.0140996)
Supplement: S3 Fig — Percentages of Ki67- and CD31-positive cells were quantified by immunohistochemistry in MB tumor samples. Five images (x 400) were captured per sample in a random manner using the Carl Zeiss-Imager.M2 microscope and quantified with the ImageJ Software. (DOCX) [file pone.0140996.s003.docx]

**S3 Fig – Quantification of Ki67 and CD31 immunolabeling.** Percentages of Ki67- and CD31-positive cells werequantified by immunohistochemistry in MB tumor samples. Five images (x 400) were captured per sample in a random manner using the Carl Zeiss-Imager.M2 microscope and quantified with the ImageJ Software.
