# Supplementary material for: Ecto-5’-Nucleotidase Overexpression Reduces Tumor Growth in a Xenograph Medulloblastoma Model
Source: PLoS One. 2015 Oct 22;10(10):e0140996. doi: 10.1371/journal.pone.0140996 (PMC4619639; doi:10.1371/journal.pone.0140996)
Supplement: S4 Fig — To determine human MB tumor growth in a nude mice in vivo model 1 x 10^6^Daoy cells were implanted by subcutaneous injection in the dorsal region of nude mice. During the tumor growth the following data were obtained: (A) Measurements of the maximum and minimum diameters of the tumor mass, which determines tumor growth (mm3). (B) Following finalization of the experiment, all animals were euthanized and the final tumor weight was determined. The values represent mean values ± SD (n = 6) for each analyzed cell group, where (*) p < 0.05 and (***) p<0.001. (DOCX) [file pone.0140996.s004.docx]

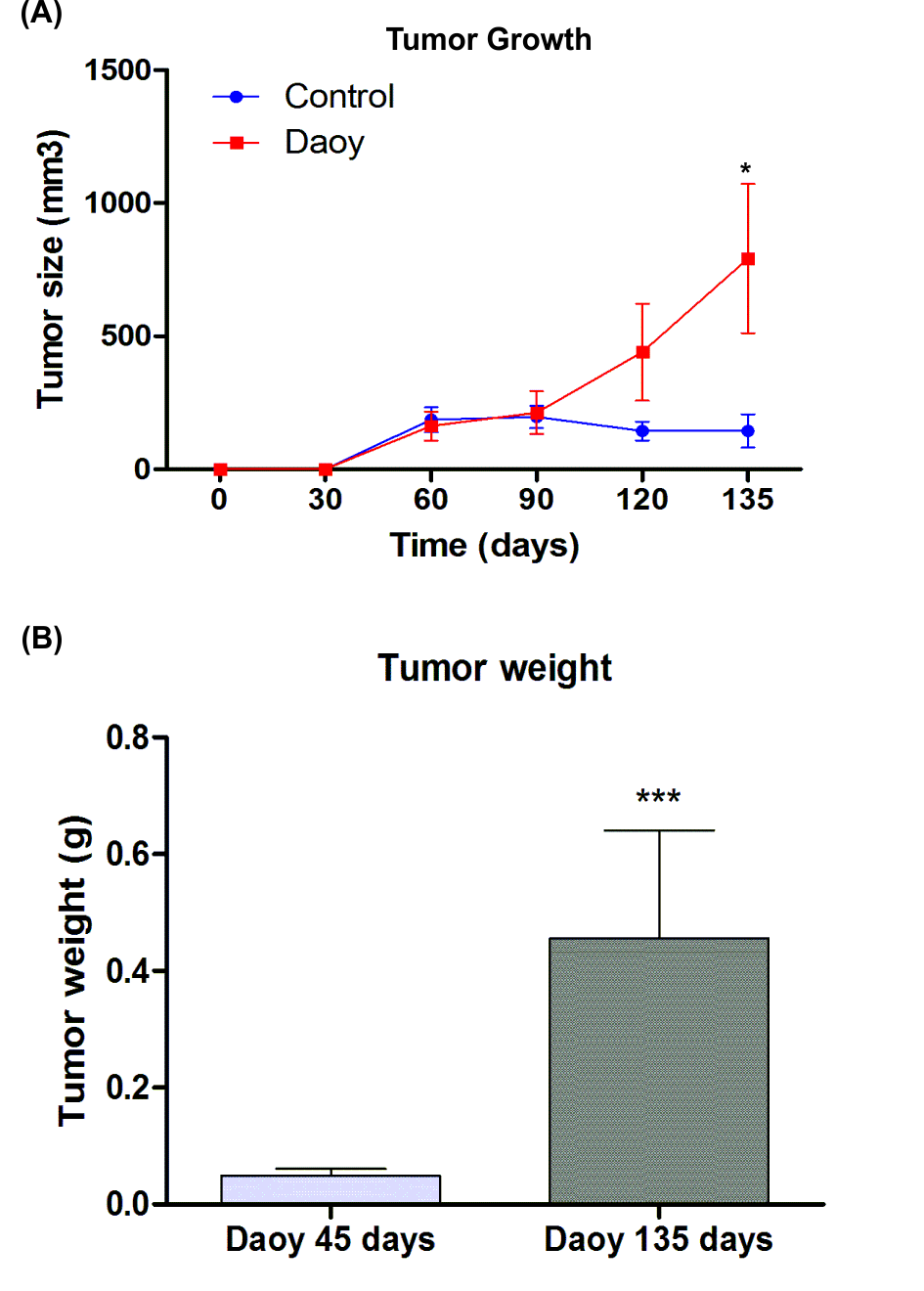


**S4 Fig – Determination of tumor growth after Daoy cell engraftment.** To determine human MB tumor growth in a nude mice *in vivo* model 1 x 10^6^Daoy cells were implanted by subcutaneous injection in the dorsal region of nude mice. During the tumor growth the following data were obtained: **(A)** Measurements of the maximum and minimum diameters of the tumor mass, which determines tumor growth (mm^3^). **(B)** Following finalization of the experiment, all animals were euthanized and the final tumor weight was determined. The values represent mean values ± SD (n=6) for each analyzed cell group, where (*) p < 0.05 and (***) p<0.001.
