# Supplementary material for: Ecto-5’-Nucleotidase Overexpression Reduces Tumor Growth in a Xenograph Medulloblastoma Model
Source: PLoS One. 2015 Oct 22;10(10):e0140996. doi: 10.1371/journal.pone.0140996 (PMC4619639; doi:10.1371/journal.pone.0140996)
Supplement: S1 Table — (DOCX) [file pone.0140996.s005.docx]

**Table S1 – Ecto-5’-NT and adenosine receptor primer sequences.**

| ***Primer sequence*** | | ***(T°C)*** | ***Fragment size (bp)*** |
| --- | --- | --- | --- |
| **CD73 F**  **CD73 R** | 5’- AGG GGT GTG GAC GTC GTG GT -3’  5’- CCC AGC AGG CAC CTC TTT GGA -3’ | 65 | 84 |
| **A1AR F**  **A1AR R** | 5’- GGA CCG CTA CCT CCG G -3’  5'-GAA GGA GAG GAT CCA GCA-3' | 55 | 100 |
| **A2AAR F**  **A2AAR R** | 5'-CAT CGT CCT CTC CCA CAC CAA-3'  5'-GTG GCT GCG AAT GAT CTT GC-3' | 58 | 100 |
| **A2BAR F**  **A2BAR R** | 5'-TTC TGG CCG TGG CAG TC-3'  5'-AGG ACA GCA ATG ACC CCT-3' | 57 | 100 |
| **A3AR F**  **A3AR R** | 5'- TCG CTG TGG ACC GAT ACT-3'  5'-ACC AGC CAG CAA AGG CC-3' | 57 | 100 |
| **GAPDH F**  **GAPDH R** | 5’- TTC TTT TGC GTC GCC AGC CG -3’  5’- ACC AGG CGC CCA ATA CGA CC -3’ | 64 | 93 |

These primers listed were used as follows. CD73 was used for RT-PCR. Primers for amplification of adenosine receptor and GAPDH sequences were used for real time PCR reactions. Melting curve analysis was performed to determine the specificity for each real-time PCR reaction.
