# Supplementary material for: Ecto-5’-Nucleotidase Overexpression Reduces Tumor Growth in a Xenograph Medulloblastoma Model
Source: PLoS One. 2015 Oct 22;10(10):e0140996. doi: 10.1371/journal.pone.0140996 (PMC4619639; doi:10.1371/journal.pone.0140996)
Supplement: S2 Table — (DOCX) [file pone.0140996.s006.docx]

**Table S2 – Histophatological characteristics of implanted Daoy MB, four months after implantation.**

|  | **Cellularity** | **Atypia** | **Necrosis** | **Mitotic index** |
| --- | --- | --- | --- | --- |
| **Daoy (n=6)** | Moderate (3/6)  Accentuated (3/6) | Accentuated (5/6) | Absent (5/6) | 17.8 |

H&E staining were analyzed by a pathologist in a blinded manner. Evaluations were made in ten randomly chosen fields (x 200) per tumor (Olympus BH-2 microscope).
